# Early inter-hospital transfer of patients with myocardial infarction without a doctor, paramedic or nurse on board: results from a French regional emergency care network

**DOI:** 10.1186/s12873-019-0280-z

**Published:** 2019-10-28

**Authors:** Sebastien Cassan, Mihaela Rata, Claire Vallenet, Philippe Fromage, Frederic Champly, Patrick Broin, Guillaume Peribois, Valerie Sierra, Cedric Lutz, Lionel Mangin, Dominique Savary, François-Xavier Ageron, Loic Belle, for the SCA-Alp Investigators

**Affiliations:** 1 Department of Cardiology and Emergency Department, Centre Hospitalier Alpes-Leman, Annemasse, France; 2 Department of Cardiology and Emergency Department, Centre Hospitalier Annecy-Genevois, Annecy, France; 3 Department of Cardiology and Emergency Department, Centre Hospitalier de Sallanches, Sallanches, France; 4 Department of Cardiology and Emergency Department, Centre Hospitalier de Thonon, Thonon, France

**Keywords:** SCA-Alp protocol, Myocardial infarction, Percutaneous coronary intervention, Transfer, France

## Abstract

**Background:**

In France, patients with acute coronary syndromes (ACS) are usually transferred from remote hospitals to percutaneous coronary intervention (PCI) centres in mobile intensive care units (MICUs) with on-board medical staff. They are then returned to the remote hospitals by MICU 48 h after PCI. However, MICU transportation and beds in a PCI centre are in short supply. Therefore, we investigated clinical outcomes among intermediate-risk ACS patients who were transferred in private ambulances without an on-board medic or paramedic; and returned to the remote hospital sooner after PCI.

**Methods:**

In the French Alps, the RESURCOR network manages ‘SCA-Alp’ transfers using strict management protocols in ambulances with trained drivers and automatic external defibrillators, but without heart rhythm monitoring. We conducted an observational retrospective study that assessed outcomes (death and emergency return to the PCI centre within 48 h) in patients transferred using SCA-Alp. Our population comprised stabilized patients with ST-segment elevation myocardial infarction (STEMI) who returned to the remote hospital within 24–48 h of PCI, and uncomplicated patients with non-ST-segment elevation myocardial infarction (NSTEMI) within 24–72 h of symptom onset who come from and returned to (‘round-trip’) the remote hospital on the day of PCI (return < 12 h after PCI).

**Results:**

Between 2010 and 2014, 101 STEMI and 490 NSTEMI patients were transferred using SCA-Alp. No adverse events occurred during transportation and no deaths were reported. Two of 591 patients (0.3% [95% confidence interval 0.1–1.4%]) experienced a stent thrombosis within 48 h of PCI that required a second urgent PCI; both were event free at 6-month follow-up.

**Conclusions:**

Inter-hospital transfer using SCA-Alp is associated with low event rates in intermediate-risk ACS patients, allowing a more streamlined use of medical facilities and freeing-up of beds in PCI centres.

## Background

Urgent percutaneous coronary intervention (PCI) for ST-segment elevation myocardial infarction (STEMI) and early revascularization for non-ST-segment elevation myocardial infarction (NSTEMI) are recommended in international guidelines [1, 2]. The main factors limiting the implementation of these recommendations are the geographic availability and the capacity of centres with cath-labs (PCI centres). One solution would be to shorten the stay of patients in PCI centres (i.e. transfer them between remote hospitals without cath-labs and PCI centres before/after the invasive procedure).

In France, following acute treatment, patients with STEMI remain in the PCI centre for 48 h before transfer. Patients with acute NSTEMI are transferred between hospitals in mobile intensive care units (MICUs), which have on-board medical/paramedic teams [3]. As both groups of patients are generally considered to be at high risk during this period, they require transfer with a doctor and nurse on board. Paul et al. [4] reported a high incidence of complications during the transportation of high-risk patients (i.e. within 12 h of acute STEMI and 24 h of complicated NSTEMI), justifying transportation with doctors on board, whereas Bawejski et al. [5] reported a low incidence of complications during the transportation of patients within 24 h of uncomplicated NSTEMI. Therefore, it may be possible to reassess the patients’ individual risk levels and adapt the type of transfer (i.e. with or without medical/paramedical staff) accordingly.

Four studies have reported on the safety of early transfer of patients with acute coronary syndromes (ACS) between remote hospitals and PCI centres [6–9]. These transfers were, however, conducted with doctors [6, 7] or nurses [8] on board and often involved low-risk patients (i.e. with unstable angina) or excluded high-risk patients with STEMI [8, 9]. The safety of return transfers of STEMI patients to remote hospitals within 48 h of their admission to the PCI centre, and the early round-trip for patients with NSTEMI within 72 h of their admission to a remote hospital, without doctors or nurses on board, remains questionable.

In our region of France, intermediate-risk ACS patients can be transferred from a remote hospital to the PCI centre (NSTEMI patients only) and back to the remote centre after PCI (NSTEMI and STEMI patients) using the ‘SCA-Alp’ protocol. This involves an ambulance equipped with an automatic external defibrillator, driven by a driver with specialist training in basic life support, but without a medic or paramedic on board. Further, patients are returned to the remote hospital within 12 h (NSTEMI) or 24–48 h (STEMI) of PCI, rather than the standard 48 h. The aim of this retrospective, observational study was to report clinical outcomes in such patients transferred using the SCA-Alp protocol.

## Methods

This was a retrospective, observational study on early transfer (without doctors or nurses on board) of intermediate-risk patients with NSTEMI or STEMI between one of seven remote hospitals in the Haute-Savoie department in France and one PCI centre.

**Fig. 1:**
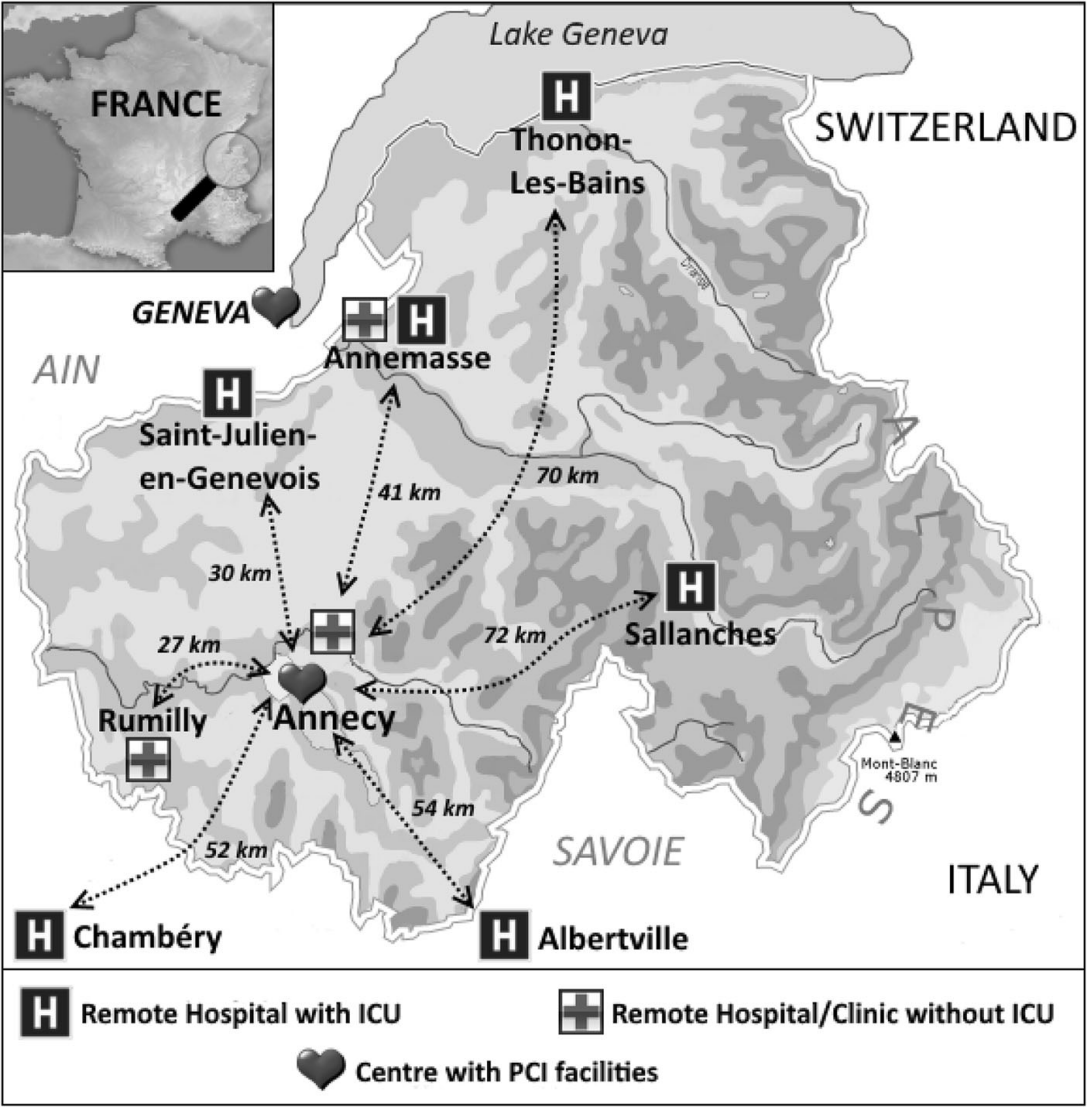
The Haute-Savoie hospital network. ICU, intensive care unit; PCI, percutaneous coronary intervention

The Haute-Savoie department is a mountainous area with approximately 800,000 inhabitants in 2015 [10]. One PCI centre (Annecy hospital) collaborates with four remote hospitals that have intensive care units (ICUs) and a MICU team and three hospitals/clinics without ICUs or a MICU team (Fig. 1). Two remote hospitals with ICUs and MICU teams (Chambéry and Albertville), located outside the Haute-Savoie department, collaborate partially with the PCI centre. The PCI centre has two cath-labs and four on-call interventional cardiologists. Eight beds are available exclusively for pre- and post-coronary intervention monitoring in a room located close to the cath-lab from 8 am to 8 pm.

In this geographic area, the RESeau des URgences CORonaires (RESURCOR) network is a coordinated regional system of care for patients with cardiac emergencies, and forms part of REseau Nord Alpin des Urgences (RENAU). The RESURCOR network disseminates protocols via pocket cards and booklets, available at a dedicated website (www.renau.org). An ongoing regional prospective registry of STEMI patients has provided data since 2002 on the acute phase of patient care [11–15].

**Fig. 2:**
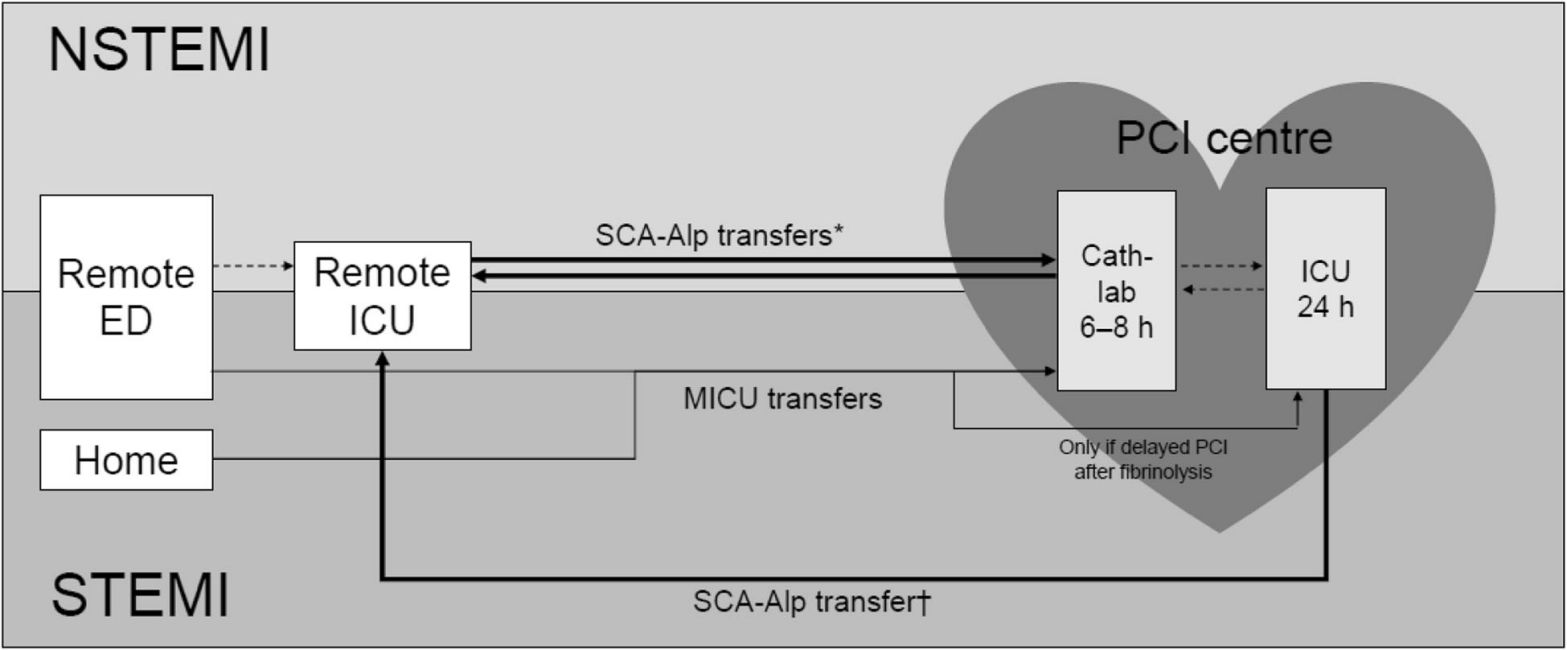
Organization of transfers between remote hospitals and the PCI centre. ED: emergency department; ICU: intensive care unit; MICU: mobile intensive care unit; NSTEMI: non-ST-segment elevation myocardial infarction; PCI: percutaneous coronary intervention; STEMI: ST-segment elevation myocardial infarction. *Only NSTEMI patients who had been stable for ≥24 h in the remote centre were eligible for SCA-Alp transfer to the PCI centre; only NSTEMI patients with optimal PCI results were eligible for SCA-Alp transfer back to the remote hospital (within 12 h of PCI). †Only STEMI patients with an optimal revascularization procedure and no recurrence of angina, acute cardiac failure, or significant ventricular arrhythmias in the 24 h following admission were eligible for SCA-Alp transfer back to the remote hospital (24–48 h after PCI)

One of the protocols of the RESURCOR network – the SCA-Alp protocol (Fig. 2) – is used to implement early transfers from remote hospitals to and from the PCI centre for intermediate-risk patients with acute NSTEMI; and early return transfers from PCI centres to remote hospitals for intermediate-risk patients with acute STEMI. SCA-Alp transfers occur in private ambulances with two basic life support-trained drivers on board and equipped with an automatic defibrillator, but without heart rhythm monitoring. Prior to the introduction of the SCA-Alp protocol, high- and intermediate-risk patients would have been transferred by MICU, with low-risk patients being transported by regular ambulance; and patients would have remained in the PCI centre for 48 h after PCI.

Patients with NSTEMI who have been admitted to an ICU in a remote hospital are transferred according to the SCA-Alp protocol to the PCI centre within 24–72 h (Fig. 2; Table 1). Patients are only eligible for SCA-Alp transfer (instead of by MICU) from the remote hospital to the PCI centre if they had been stable for at least 24 h in the remote centre (i.e. no acute cardiac failure condition, no underlying ventricular arrhythmia, and no ST-segment depression > 3 mm in ≥2 leads). Patients who underwent PCI were only eligible for SCA-Alp transfer back to the remote centre within 12 h of the procedure if the PCI result was optimal. Otherwise, they remained in the PCI centre.

**Table 1:** Recommended transfer types

	Low-risk patients	Intermediate-risk patients	High-risk patients
NSTEMI			
Remote hospital to PCI centre	Ambulance if uncomplicated;^a^ > 72 h after admission	SCA-Alp if uncomplicated;^a^ 24–72 h after admission	MICU if uncomplicated;^a^ < 24 h after admission; or complicated
Return to remote hospital	Ambulance if uncomplicated;^a^ > 12 h after successful PCI	SCA-Alp if uncomplicated;^a^ < 12 h after successful PCI	
STEMI			
Remote hospital (or home) to PCI centre	MICU	MICU	MICU
Return to remote hospital	Ambulance if uncomplicated;^b^ > 48 h after PCI	SCA-Alp if uncomplicated;^b^ 24–48 h after successful PCI	Remain in PCI centre if complicated or < 24 h

*MICU* mobile intensive care unit, *NSTEMI* non-ST-segment elevation myocardial infarction, *PCI* percutaneous coronary intervention, *SCA-Alp* ambulance with trained drivers and an automatic defibrillator, *STEMI* ST-segment elevation myocardial infarction

^a^No acute cardiac failure condition, no underlying ventricular arrhythmia, and no ST-segment depression > 3 mm in ≥2 leads

^b^No cardiac failure, no recurrent angina, no significant ventricular arrhythmia

Patients with STEMI diagnosed in a remote hospital are transferred straight to the PCI centre in a MICU, where a primary or rescue angioplasty after thrombolysis may be performed (Fig. 2; Table 1). After PCI, intermediate-risk patients are transferred back to the remote hospital (closest to the patient’s home) within 24–48 h using a SCA-Alp transfer. This only applies to patients with an optimal revascularization procedure and no recurrence of angina, acute cardiac failure, or significant ventricular arrhythmias in the 24 h following admission. High-risk STEMI patients (i.e. non-optimal revascularization, recurrence of angina, acute cardiac failure, or significant ventricular arrhythmias within 24 h after PCI) remain at the PCI centre.

Low-risk patients who are returned to the remote hospital > 48 h after PCI are not eligible for the SCA-Alp protocol. Instead, they are discharged or transferred back to the remote hospital by regular ambulance.

### Study population

The population comprised consecutive patients admitted to the PCI centre in Annecy from January 2010 to December 2014 with an initial diagnosis of acute STEMI or NSTEMI. Patients with STEMI were eligible for inclusion if they had a transfer according to the SCA-Alp protocol to a remote hospital 24–48 h after admission to the PCI centre in Annecy. Patients with NSTEMI were eligible for inclusion if they were admitted to a remote hospital, transferred using the SCA-Alp protocol to the PCI centre in Annecy within 72 h, and transferred back under the same protocol within 12 h of admission to the PCI centre in Annecy.

### Data collected and study endpoints

Descriptive data were derived from the RESURCOR STEMI registry and the cath-lab database. Troponin concentrations were obtained from the remote hospital lab databases. In-hospital mortality data were derived from each hospital’s database; retransfer from a remote hospital to the PCI hospital for a coronary angiogram within 48 h of the return to the remote hospital was derived from the cath-lab database. The medical records (from the PCI centre and the remote hospitals) of all patients who underwent PCI were routinely examined by a study technician in charge of the cath-lab database and data on endpoints were collected.

The aim of this study was to determine the rate of adverse events associated with the SCA-Alp approach, to ascertain whether this approach is safe. The primary endpoints of this study were death within 48 h of return to the remote hospital (including during transfer) and the requirement for retransfer back to the PCI centre within 48 h for a coronary angiogram. Secondary endpoints were in-hospital stroke, Thrombolysis In Myocardial Infarction (TIMI) major bleeding, major haematoma at the puncture site (defined as the need for a transfused haemostatic agent or surgical intervention), and intrastent thrombosis (or reocclusion of the dilated lesion) > 48 h after PCI. These endpoints were only available for patients who underwent PCI.

### Statistical analysis

Values are expressed as mean ± standard deviation (SD) for continuous variables and as counts and percentages for categorical variables. The 95% confidence interval for the primary outcome was calculated using the Wilson method. All analyses were conducted in Microsoft Excel.

## Results

**Fig. 3:**
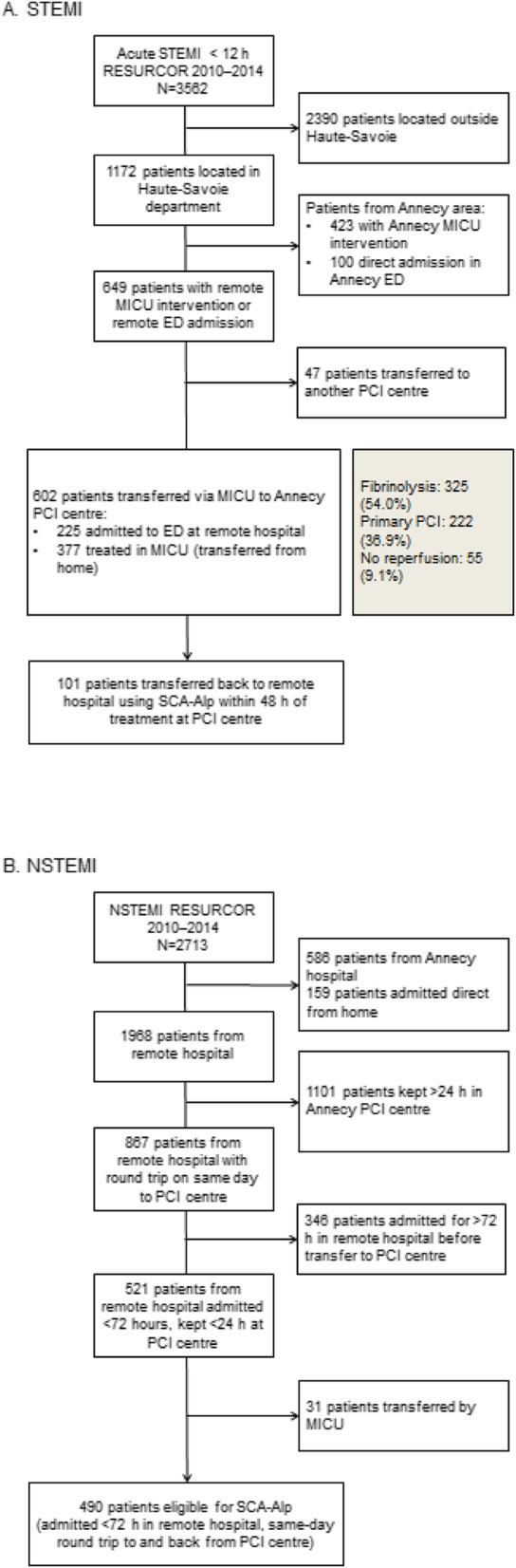
Flow charts of patients with **a** STEMI and **b** NSTEMI. ED: emergency department; NSTEMI: non-ST-segment elevation myocardial infarction; STEMI: ST-segment elevation myocardial infarction

During the study period, 1172 patients with STEMI were treated in the Annecy PCI centre. Of these, 602 patients were transferred immediately (via MICU) from the emergency department at a remote hospital to the PCI centre (*n* = 225) or arrived after initial treatment in a MICU (from home) from a remote area (*n* = 377). Just over half (54.0%) were treated with thrombolysis and 36.9% were admitted for primary PCI. One hundred and one of these patients were returned to their remote hospital using the SCA-Alp protocol within 48 h (Fig. 3a).

During the same period, 1968 patients with an initial diagnosis of NSTEMI were transferred from a remote hospital to the Annecy PCI centre, of which 867 were transferred using the SCA-Alp protocol and transferred back within 12 h. A total of 490 patients initially stayed < 72 h in the remote hospital before transfer to the PCI centre and were transferred back using the SCA-Alp protocol within 12 h (Fig. 3b) (198 patients were transferred within 24 h, 168 between 24 and 48 h, and 124 between 48 and 72 h).

Patient baseline characteristics, initial management, and angiographic characteristics are detailed in Tables 2 and 3. All patients were treated with a P2Y_12_ inhibitor before arrival at the cath-lab. Access was radial for most patients (94.6%).

**Table 2:** Patient baseline characteristics

	STEMI (*n* = 101)	NSTEMI (*n* = 490)
Age, years	56 ± 14	65 ± 13
Men	84 (83.2)	357 (72.9)
Medical history		
Hypertension	27 (26.7)	225 (45.9)
Diabetes mellitus	12 (11.9)	89 (18.2)
Smoking (current or past)	53 (52.5)	162 (33.1)
Dyslipidaemia	20 (19.8)	156 (31.8)
Peripheral artery disease	1 (1.0)	14 (2.9)
Stroke	2 (2.0)	14 (2.9)
Previous coronary artery disease		
PCI	7 (6.9)	70 (14.3)
CABG	1 (1.0)	8 (1.6)
Myocardial infarction	9 (8.9)	47 (9.6)
Initial clinical presentation		
SBP at admission, mmHg	147.5 ± 31.8	
Anterior myocardial infarction	36 (35.6)	
Lateral myocardial infarction	9 (8.9)	
Inferior myocardial infarction	56 (55.4)	

Data presented as mean ± SD or *n* (%)

*CABG* coronary artery bypass graft, *NSTEMI* non-ST-segment elevation myocardial infarction, *PCI* percutaneous coronary intervention, *SBP* systolic blood pressure, *STEMI* ST-segment elevation myocardial infarction

**Table 3:** Initial treatment and angiographic characteristics

	STEMI (*n* = 101)	NSTEMI (*n* = 490)
Initial management		
Primary MICU involvement	79 (78.2)	–
Initial remote ED admission and immediate MICU transfer^a^	22 (21.8)	–
Medication before arrival in the cath-lab		
Aspirin	95 (94.1)	473 (96.5)
Clopidogrel	63 (62.4)	310 (63.3)
Prasugrel	14 (13.9)	10 (2.0)
Ticagrelor	24 (23.8)	170 (34.7)
Unfractionated heparin	15 (14.9)	191 (39.0)
Low-molecular-weight heparin	71 (70.3)	292 (59.6)
Bivalirudin	15 (14.9)	7 (1.4)
GP IIb/IIIa inhibitor	30 (29.7)	98 (20.0)
Interventional characteristics		
Characteristics of CAD		
Single-vessel disease	69 (68.3)	258 (52.7)
Two-vessel disease	25 (24.8)	119 (24.3)
Three-vessel disease	7 (6.9)	57 (11.6)
No significant CAD	0	56 (11.4)
Emergency primary coronary angiogram	43 (42.6)	
(Primary) PCI	33/43 (76.7)	274/490 (55.9)
Fibrinolysis	58 (57.4)	
Rescue coronary angiogram after fibrinolysis	27 (26.7)	
Rescue PCI after fibrinolysis	20/27 (74.1)	
Delayed coronary angiogram after fibrinolysis	31 (30.7)	
Delayed PCI after fibrinolysis	29/31 (93.5)	
Delayed bypass	2 (2.0)	26 (5.3)
Medical treatment only (no PCI or CABG)	17 (16.8)	190 (38.8)
Stent implantation	75 (74.3)	250 (51.0)
Balloon only (no stent)	7 (6.9)	24 (4.9)

Data presented as *n* (%), *n*/*N* (%), or mean ± SD

*CABG* coronary artery bypass graft, *CAD* coronary artery disease, *ED* emergency department, *GP* glycoprotein, *MICU* mobile intensive care unit, *NSTEMI* non-ST segment elevation myocardial infarction, *PCI* percutaneous coronary intervention, *SD* standard deviation, *STEMI* ST segment elevation myocardial infarction

^a^Patients who arrived at the remote hospital using their own transport

Ten of the 43 patients with STEMI admitted for an emergency angiogram and without previous fibrinolysis did not undergo PCI: four of these patients had a very narrow infarct-related artery (three had a final TIMI flow of 0 and one had a final TIMI flow of 2) and six had an initial TIMI flow of 3 and a non-significant lesion and/or the artery was too small to be dilated. Table 4 shows the initial TIMI flow distribution of the 43 patients admitted for an emergency angiogram without previous fibrinolysis and the final TIMI flow of the 82 patients treated with PCI (33 primary PCI, 20 rescue PCI after failed thrombolysis, and 29 treated with delayed PCI after successful thrombolysis).

**Table 4:** Angiographic TIMI scores for patients with STEMI

TIMI score	Initial^a^ (*n* = 43)	Final^b^ (*n* = 82)
0	28 (65.1)	3 (3.7)
1	6 (14.0)	0
2	0	1 (1.2)
3	9 (20.9)	78 (95.1)

Data presented as *n* (%)

*PCI* percutaneous coronary intervention, *STEMI* ST-segment elevation myocardial infarction, *TIMI* Thrombolysis In Myocardial Infarction

^a^Only for those who underwent primary PCI

^b^For primary, rescue, or delayed PCI with effective intervention

Most patients with STEMI (90/101, 89.1%) or NSTEMI (467/490, 95.3%) were transferred from or to the four main remote hospitals with an ICU. The mean maximum troponin concentration among NSTEMI patients was 1.69 ± 7.8 μmol/L and the overall mean length of hospitalization in the remote hospital was 5.0 ± 3.2 days.

### Outcomes

No deaths or other major events were reported during transportation; and no deaths occurred during the 48 h after transfer to the remote hospital. Two of 591 patients (0.3% [95% confidence interval 0.1–1.4%]) had to be transferred back to the PCI centre for repeat coronary angiography within 2 days of their primary PCI (Table 5). The first was a 36-year-old man admitted with anterior STEMI. Treatment with clopidogrel, aspirin, and bivalirudin was started before arrival at the cath-lab. The emergency coronary angiogram showed an occlusion with TIMI 0 flow of the left anterior descending artery. After thrombus aspiration, TIMI 3 flow was restored and the lesion was not significant. The patient was not stented and bivalirudin treatment was switched to low-molecular-weight heparin 2 h after the procedure. The patient was transferred to a remote hospital the following day, but experienced chest pain with ST-segment elevation 48 h after admission. He was immediately transferred in a MICU for an emergency coronary angiogram, which showed reocclusion of the infarct-related artery. Treatment with a glycoprotein IIb/IIIa inhibitor was administered in the cath-lab and a stent was implanted. The patient was event free at 6-month follow-up.

**Table 5:** Clinical outcomes

	STEMI (*n* = 101)	NSTEMI (*n* = 490)
Primary endpoints		
Death (in the first 48 h post PCI)	0	0
Retransfer to PCI centre for second emergency coronary angiogram (< 48 h after the cath-lab)^a^	1 (1.0)	1 (0.2)
Secondary endpoints		
Stroke	0	0
Major haematoma at the puncture site	1 (1.0)	0
Major bleeding complication	0	1 (0.2)
Stent thrombosis > 48 h after PCI	0	1 (0.2)

Data presented as *n* (%)

*NSTEMI* non-ST-segment elevation myocardial infarction, *PCI* percutaneous coronary intervention, *STEMI* ST-segment elevation myocardial infarction

^a^For suspicion of stent thrombosis or dilated artery reocclusion in the absence of a stent placement or dilated artery reocclusion in the absence of a stent

The second case was a 74-year-old man who was admitted to a remote hospital with NSTEMI and was treated with clopidogrel, aspirin, and low-molecular-weight heparin. He was transferred according to the SCA-Alp protocol to the PCI centre 48 h after admission. A 70% stenosis was diagnosed in the mid left descending artery and a stent was implanted with a good result. He was transferred back to the remote hospital 4 h after PCI. He suffered an anterior STEMI 24 h after his return and was immediately transferred via MICU for an emergency coronary angiogram, which showed a stent thrombosis. This was treated with a glycoprotein IIb/IIIa inhibitor and aspiration, and another stent was implanted downstream due to a possible occlusive dissection. The patient was event free at 6-month follow-up.

One patient experienced a radial haematoma that required surgical intervention the day after transfer to the remote hospital. One patient – who was receiving a glycoprotein IIb/IIIa inhibitor after the coronary angiogram – experienced a cerebral haematoma a few hours after his return to the remote hospital, but did not require surgical intervention and had no neurological disability. One patient had a STEMI due to a stent thrombosis 8 days after PCI, requiring emergency primary PCI.

## Discussion

This retrospective study shows the feasibility of a protocol for early return to a non-interventional hospital after PCI for selected intermediate-risk patients with an ACS. No adverse events occurred during transportation without doctors or nurses on board during return transfer 24–48 h after PCI of patients with STEMI or for same-day round trips for patients within 24–72 h of NSTEMI. No deaths were reported and only two of 591 patients needed to be transferred back to the PCI centre within 48 h (in a MICU) for another PCI due to stent thrombosis. The SCA-Alp approach frees up beds in PCI centres, which are in increasing demand. Performing these transfers without doctors or nurses on board allows a more streamlined use of medical facilities, but requires a well-organized system with well-targeted patients, shared protocols and trained staff, as followed in our SCA-Alp protocol.

Comparison of our results with previous reports on early return transfers is difficult due to the heterogeneity in the risk level of ACS patients. Some studies have included high-risk patients with acute STEMI [4, 6, 7], while others have included lower-risk patients with NSTEMI > 72 h previously or unstable angina [8, 9]. Also, some transfers were performed with doctors or paramedics on board and others without. However, our results are concordant with these reports regarding the risk of stent thrombosis (0–2.2%) and in-hospital death (0–3.2%) [6–9].

As MICUs are few in number and expensive, we hypothesised that many high-risk patients might be classified as being at intermediate risk for inter-hospital transfer, and hence be suitable for SCA-Alp transfer. We can define high-risk patients as those with acute STEMI, complicated patients, and those who have had an uncomplicated STEMI or NSTEMI within the last 24 h. Such patients require MICU transportation. Low-risk patients are those with uncomplicated STEMI > 48 h ago or uncomplicated NSTEMI > 72 h ago. Intermediate-risk patients can therefore be defined as those with uncomplicated STEMI within the last 24–48 h or uncomplicated NSTEMI within the last 24–72 h. The results of our study suggest that these patients can be safely transferred using a protocol such as SCA-Alp. The risk level of patients with uncomplicated NSTEMI within the last 24 h is unclear, but they may be considered at intermediate risk, as Bawejski et al. [5] reported no complications requiring medical intervention among 93 such patients transported with doctors or nurses on board. Moreover, despite our protocol criteria, 198 patients with NSTEMI were transferred within 24 h by SCA-Alp in our study, without any reported complications. Of course, risk levels may be better defined using scoring systems such as the Global Registry of Acute Coronary Events (GRACE) [16]. This may provide a better risk estimation, and the use of GRACE could be examined in a future study.

Our approach addresses an important public health issue as most of our patients came from remote areas (602/1172 [51.3%] STEMI and 1968/2554 [77.1%] NSTEMI), and there are not enough doctors to perform such MICU transfers. The SCA-Alp protocol is based on a strong network and shared practices regarding cardiovascular diseases in our region.

Reocclusion occurred in two of our patients, one of whom was treated with bivalirudin, which increases the risk of acute stent thrombosis [17, 18]. Our inclusion period was also transitional in terms of antiplatelet therapy, and perhaps these stent thromboses would have been avoided with the use of the newest P2Y_12_ inhibitors, ticagrelor and prasugrel. Indeed, PCI techniques continue to evolve and the intervention is becoming safer, allowing earlier return of patients, with almost systematic use of radial access for the coronary angiogram (> 94%), which is known to reduce bleeding risk [19].

### Limitations

This retrospective observational study is subject to the usual limitations of such analyses. Some events may not have been reported by the remote hospitals, and the reporting of complications was limited to patients who had a successful PCI (with the exception of the primary endpoints, which were known for all patients). It is possible that some events (e.g. stent thrombosis in an elderly patient) did not result in their transfer back to the PCI centre. However, the examination of remote hospital medical records of patients who had undergone PCI did not reveal any such unreported events. In view of the study aims, we focused on outcomes that would have proven the failure of early return. We acknowledge that other complications, such as stroke or bleeding events, are similarly treated in remote hospitals. Patients were not under continuous rhythm monitoring during transportation, so we cannot exclude the occurrence of minor complications that did not require medical intervention [5]. As a retrospective study, some patients who would have been eligible for the SCA-Alp protocol were not included for reasons that remain unknown. In addition, we cannot confirm that the SCA-Alp protocol was followed precisely. For example, 198 patients with NSTEMI < 24 h previously were included, who were not eligible according to the protocol.

## Conclusions

The results of this retrospective observational study suggest that the early transfer of intermediate-risk ACS patients between PCI centres and remote hospitals – without doctors, paramedics, or nurses on board – is feasible, and is associated with low event rates.

## Appendix

**Table:** Investigators and remote hospital destinations

	Investigator	STEMI (*n* = 101)	NSTEMI (*n* = 490)
Annemasse hospital	Claire Vallenet	35 (34.7)	200 (40.8)
Sallanches hospital	Frederic Champly	22 (21.8)	83 (16.9)
Thonon hospital	Guillaume Peribois	21 (20.8)	126 (25.7)
Saint Julien hospital	Cedric Lutz	12 (11.9)	58 (11.8)
Albertville hospital	Marc Haesevoets	5 (5.0)	5 (1.0)
Chambery hospital	Pascal Usseglio	6 (5.9)	8 (1.6)
Annemasse clinic	Christian Curvat	0	7 (1.4)
Argonay clinic (Annecy)	Dominique Savary	0	2 (0.4)
Rumilly hospital	Dominique Savary	0	1 (0.2)

Data are presented as *n* (%)

*NSTEMI* non-ST-segment elevation myocardial infarction, *STEMI* ST-segment elevation myocardial infarction

## Abbreviations

ACS: Acute coronary syndromes
ED: Emergency department
ICU: Intensive care unit
ICU: Intensive care unit
MICU: Mobile intensive care unit
NSTEMI: Non-ST-segment elevation myocardial infarction
PCI: Percutaneous coronary intervention
PCI: Percutaneous coronary intervention
RENAU: REseau Nord Alpin des Urgences
RESURCOR: RESeau des URgences CORonaires
SD: Standard deviation
STEMI: ST-segment elevation myocardial infarction
TIMI: Thrombolysis In Myocardial Infarction
